# The influence of geographical location on moisture distribution in wood cross sections: a numerical simulation study using Austria as an example

**DOI:** 10.1186/s10086-024-02147-z

**Published:** 2024-07-30

**Authors:** Florian Brandstätter, Maximilian Autengruber, Markus Lukacevic, Josef Füssl

**Affiliations:** TU Wien, Institute for Mechanics of Materials and Structures, Karlsplatz 13, 1040 Vienna, Austria

**Keywords:** Coupled moisture transport, Heat mass transfer, Wood, Timber, Moisture content distribution, Moisture-induced stresses, Location, ONR CEN/TS 19103

## Abstract

Wood constantly interacts with the surrounding, locally varying climate, leading to changes in the moisture content. Advanced simulation tools can predict the two-dimensional moisture distributions caused by these changing climate conditions within wood cross sections over time. However, there is a notable absence of systematic simulation results for diverse climatic conditions and various wood cross sections. This study seeks to bridge this gap in research. Here, we present moisture fields in three solid timber and three glued laminated timber cross sections in Austria and show the effect of the location and the altitude on the moisture content distribution. The results reveal decreasing influence of the location on the moisture content development with increasing cross section size, and primarily the altitude affecting the moisture content. In addition, the results are compared with the standard for the design of timber–concrete composite structures (ONR CEN/TS 19103), revealing appropriate values in most of the cases. Only for cross sections with a width of 14 cm and larger, assigned to a specific region, the standard value is assumed underestimated. Furthermore, the distribution of moisture gradients, which are related to the crack depth development, are analyzed for Austria, demonstrating the influence of mountain areas in the moisture gradient development.

**Supplementary Information:**

The online version contains supplementary material available at 10.1186/s10086-024-02147-z.

## Introduction

It is well known that the material properties of wood are affected by its moisture content *u* and temperature as well as their changes, which is why they have to be considered when designing timber constructions. Besides, anisotropy and inhomogeneity characterize the material, since wood consists of cells running mostly parallel to the trunk with varying cell structure and growth patterns. Whereas inhomogeneity with respect to annual rings can be neglected as a sufficient number of annual rings create suitable conditions for homogenization, anisotropy must be considered due to the wooden cell arrangement, which causes a directionality in load application (longitudinal, radial and tangential). In addition, due to the hygroscopic properties of wood, the cell wall can adsorb and desorb moisture out of the surrounding environment changing the volume. Due to non-uniform direction-dependent hygro-expansion coefficients, strains caused by changes in the moisture content result in eigenstresses, which can cause crack initiation and propagation.

To address the impacts of moisture and temperature when designing timber constructions, the standard EC 5 [1] defines three so-called service classes. The assignment to a class depends on the surrounding relative humidity (RH) level with an assumed reference temperature of 20 $^{\circ }$C. Furthermore, EC 5 specifies that the moisture content averaged over the cross section $u_{\text{avg,cs}}$ of most softwoods is below one out of two levels. These values are the equilibrium moisture content levels resulting from the relative humidity thresholds according to the adsorption isotherm at 20 $^{\circ }$C. Service class 1 is assigned when structures are subjected to a climate where the RH exceeds 65% only a few weeks a year, and $u_{\text{avg,cs}}$ is below 12% at any time. This specific climate condition (65% RH at 20 $^{\circ }$C) serves as the reference for defining wood-specific values in EC 5. In case of service class 2, the maximum RH only exceeds 85% for a couple of weeks a year, and $u_{\text{avg,cs}}$ is constantly below 20%. Service class 3 must be assigned when $u_{\text{avg,cs}}$ exceeds 20%, a threshold conducive to fungi and mold growth as well as insect infestation, thus compromising the structural integrity and durability of timber elements [2].

Several scientists have investigated moisture’s impact on wood. In a previous study, Autengruber et al. [3] analyzed moisture fields and crack patterns in 18 wooden cross sections under a representative climate to examine the influence of the cross section size on moisture development. Building upon this research, we aim to study the effect of varying climates at various locations on wooden cross sections, as several works indicate the significance of the location on the moisture content [4–7]. Schiere et al. [4] compared the average moisture content of measurements obtained from timber constructions (e.g., bridges and ice rinks) with the determined moisture content based on the RH and temperature data from 107 meteorological stations in Switzerland. The measured data showed a wide range in average RH levels, indicating large fluctuations in the moisture content at different locations affecting wood properties. Franke et al. [5] presented various recommendations for the design, assembly, and monitoring of timber constructions considering their moisture content level. In addition, the relevance of the location-specific climate throughout Switzerland is highlighted, pointed out by the distribution of the equilibrium moisture content, which is between 12 and 20%, and the yearly fluctuations of the moisture content at the cross section’s surface (between 7 and 10%). Fragiacomo et al. [6] examined the influence of three different climates distributed across Europe on moisture-induced stresses in timber cross sections. It was shown that Northern climates (measurements from Rovaniemi) cause higher surface tensile stresses compared to Southern climates (measurements from Lisbon). The standard ONR CEN/TS 19103 [7] defines common rules for the structural design of timber–concrete composite structures, in which Europe is divided into climate regions. Depending on the climate region and cross section size, values considering the yearly variation of the moisture content averaged over the cross section are assigned, which are required to determine the inelastic strain caused by swelling or shrinkage of timber.

Variations in climate cause changes in the moisture content, which can lead to cracks. Dietsch et al. [8] showed that moisture-induced cracks in large-span timber constructions can be related to moisture content gradients, and Autengruber et al. [3] pointed out that differences in the moisture content between the center and the surface of the cross section were at maximum when the deepest cracks occurred. Brandstätter et al. [9] presented a relation between moisture gradient and crack depth, enabling the estimation of crack depth development in indoor climate conditions.

The existing body of literature and research allows us to draw two key conclusions. First, the varying climatic conditions significantly influence the depth of potential cracks in wooden components. Second, advanced simulation tools now enable the prediction of two-dimensional moisture distributions within wood cross sections over time, influenced by the surrounding climate. However, there is a notable absence of systematic simulation results for diverse climatic conditions and various wood cross sections. This study seeks to bridge this gap in research. Simulation offers a more efficient and rapid method for gathering detailed information about moisture distribution in cross sections compared to on-site monitoring. Consequently, these simulations are invaluable for creating comprehensive databases that contribute to general knowledge and assist in the ongoing development of standards.

## Materials and methods

In this section, the models to describe the moisture and heat transport mechanisms and to simulate fracture in wood (Norway spruce) are introduced. In addition, the geometries of the investigated cross sections are presented and the climate data used for the simulations examined.

### Mathematical model for moisture transport in wood

Multi-Fickian transport models have frequently been used to simulate moisture transport in wooden cross sections, further developed by several authors [10–15]. It describes the diffusion processes of bound water in the wooden cells and water vapor in the lumen, coupled by the sorption rate under consideration of a hysteresis effect. In addition, an energy change term considers the heat transfer. These processes can be described by differential equations, which are presented in more detail in [3, 16–18]. It is assumed that the wooden cross sections are protected from rain and the cell walls are never fully saturated preventing the occurrence of free water.

To determine the moisture flux, the finite element software *Abaqus* [19] is used, where subroutines enable the implementation of the multi-Fickian transport model. The cross sections to be analyzed are discretized by brick-type elements with linear interpolation functions and the modified Newton method is used to solve the equations, which define the transport model.

#### Initial conditions

To simulate post-production conditions, the initial relative humidity is set to 65% and the initial temperature is 293.15 K, resulting in an initial water vapor concentration of $0.011235\,{\text {kg/m}}^3$, and an initial bound water concentration of $49.25\,{\text {kg/m}}^3$ (11.73% *u*) considering the adsorption isotherm at 293.15 K, respectively.

### Model for fracture in wood

To determine crack initiation and propagation, extended finite element method (XFEM) simulations are performed with the results of the moisture simulations as loading. The extended element method allows for considering discontinuities, such as cracks, in finite element calculations by enriching elements with further degrees of freedom and special functions to determine the displacement field. The computation of the moisture-induced stresses in each integration point for each increment is based on a linear elastic material model, and the non-uniform directional expansion coefficients are given in Table S1 in Supplementary material. A multisurface failure criterion defines the strength of wood, where exceeding causes cracking.

**Fig. 1:**
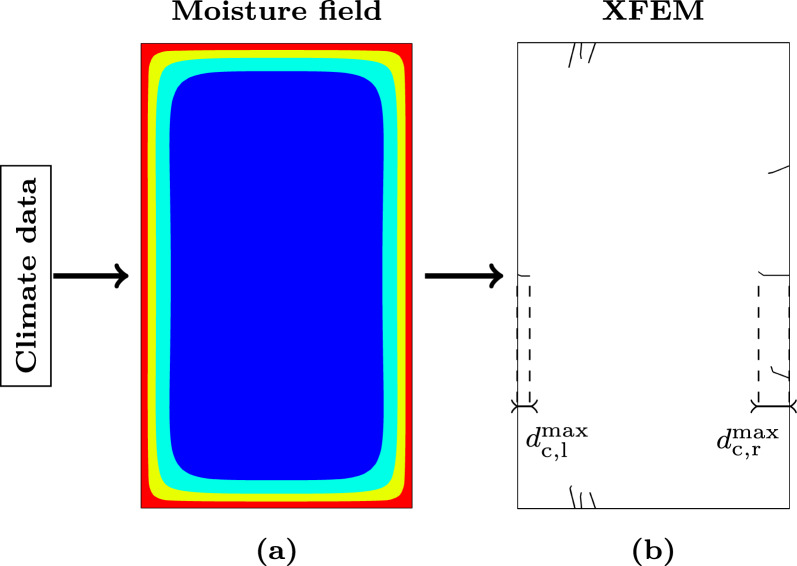
Description of the crack development process: **a** First, moisture fields were simulated for various cross sections with climate data as loading. **b** In a subsequent XFEM simulation, where the moisture fields served as loading, stresses were determined. If these exceed the limits of the multisurface failure criterion, which is evaluated in each integration point, cracks initiate or propagate

As the deepest cracks affect the load-bearing capacity most, the maximum crack depth development is examined. Therefore, the crack depth $d_{\text{c}}$ is determined for both cracks starting at the left and right edge of the cross sections, with $d_{\text{c}}$ as the perpendicular distance from the surface to the crack tip. The sum of the deepest cracks from both edges ($d_{\text{c,l}}^{\text{max}}$ and $d_{\text{c,r}}^{\text{max}}$) is defined as the maximum total crack depth $d_{\text{c}}^{\text{max}}$ (see Fig. 1).

#### Material properties

For the simulations, the wood type is defined as Norway spruce (*Picea abies*) with a clear wood dry density of $420\,\text{kg/m}^3$. To consider the moisture-dependent material properties, the continuum micromechanics material model of Hofstetter et al. [20] is used, where the elasticity tensor components for moisture content levels between 3 and 30% were predetermined (see Table S2 in Supplementary material).

#### Multisurface failure criterion

To simulate fracture in wood, a multisurface failure criterion is used [21–24], which considers wood’s irregular structure at the level of annual rings (late- and earlywood). The development of the used criterion is described in Tsai and Wu [25] and resulted in the definition of eight Tsai-Wu failure surfaces:

1 $$\begin{aligned} f_\text{i}^{\text{cw}} \left( \mathbf {\sigma } \right)& = a_{\text{LL,i}} \, \sigma _{\text{LL}} + a_{\text{RR,i}} \, \sigma _{\text{RR}} + a_{\text{TT,i}} \, \sigma _{\text{TT}} + b_{\text{LLLL,i}} \, \sigma ^2_{\text{LL}} + b_{\text{RRRR,i}} \, \sigma ^2_{\text{RR}} \\ & \quad + b_{\text{TTTT,i}} \, \sigma ^2_{\text{TT}} + 2b_{\text{RRTT,i}} \, \sigma _{\text{RR}} \, \sigma _{\text{TT}} + 4b_{\text{LRLR,i}} \, \tau ^2_{\text{LR}} + 4b_{\text{RTRT,i}} \, \tau ^2_{\text{RT}} + 4b_{\text{TLTL,i}} \, \tau ^2_{\text{TL}} \le 1. \end{aligned}$$

This enables brittle (cracking) and ductile (plastic) failure behavior. In case of brittle material failure, the corresponding failure surface defines the crack initiation or propagation orientation. Depending on the material orientation, strength limits vary (longitudinal 56 MPa, radial 5 MPa and tangential 2 MPa). Although moisture influences wood strength, it is assumed that changes in *u* do not affect the failure surfaces. In case of crack initiation and propagation, the corresponding regions are characterized by an moisture content of about 12%, which is the reference level for the criterion. Thus, the error is negligibly small.

### Geometries

**Fig. 2:**
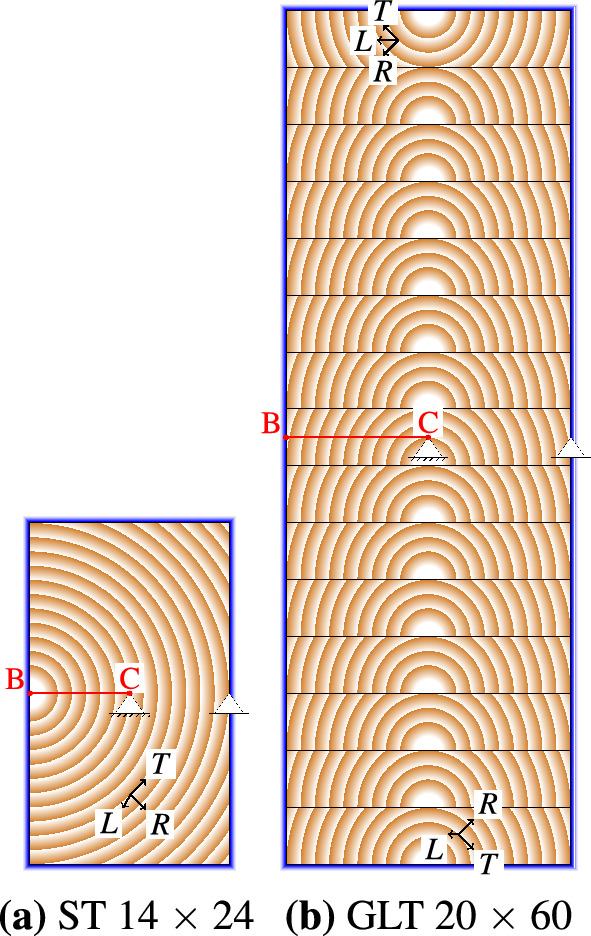
Illustration of the pith locations, the definition of the local coordinate system as well as of the geometric boundary conditions, exemplified for one ST (**a **ST $14 \times 24$) and one GLT (**b** GLT $20 \times 60$) cross section (width $\times$ height, given in cm). The path from the boundary (B) to the center (C) is displayed and the blue frame shows on which edges the boundary conditions are applied during the moisture field simulation

To highlight geometry-based differences, six different cross section sizes are used for the simulations: three solid timber type (ST) cross sections (ST $5\times 8$, ST $8 \times 16$ and ST $14 \times 24$, given in cm) and three glued laminated timber (GLT) type cross sections (GLT $16 \times 60$, GLT $20 \times 60$ and GLT $24 \times 60$), with a lamella height of 4 cm. As shown in [3], the location of the pith influences the moisture-induced stresses, which is why the pith of the ST cross sections is positioned in the middle of the left edge and the piths of the GLT lamellas are in the middle of the bottom side to induce the largest stress possible (see Fig. 2). As usual in common production standards, the lamella at the top is flipped. Since the diffusion coefficients in radial and tangential direction of bound water and water vapor [13, 26] are assumed equal, the moisture transport is not influenced by the pith location.

Volkmer et al. [27] showed that the adhesives UF (urea-formaldehyde) and MUF (melamine urea-formaldehyde) used in timber production minimally decrease the permeance of water vapor. Thus, it is assumed that for the GLT cross sections the glue lines do not influence the moisture transport and are thus neglected, as presented in Autengruber et al. [3].

Depending on the cross section, the number of finite elements, as well as their width and height varies (see Table 1). For all cross sections, a non-uniform distribution of the elements was chosen. For the ST cross sections, a double-bias for all edges was defined, whereas for the GLT cross sections, all edges of each lamella are seeded as double-bias. Thus, the height and width of the elements are minimal close to the edge’s ends, and increase towards the middle of the edge. In longitudinal direction, there is one element with a thickness of 1 mm. Therefore, plane strain conditions are assumed. In the initial state, no eigenstresses, e.g., caused by production processes, are present.

**Table 1:** Number of elements as well as height and width range of elements for all cross sections

	ST			GLT		
	Width $\times$ height (cm)					
	$5\times 8$	$8 \times 16$	$14\times 24$	$16 \times 60$	$20\times 60$	$24 \times 60$
Number of elements	$20 \times 33$	$18 \times 40$	$26 \times 47$	$24 \times 105$	$30 \times 105$	$36 \times 105$
Width of elements (mm)	1–5	1–11	1–15	2–15	2–15	2–15
Height of elements (mm)	1–5	1–10	1–15	2–14	2–14	2–14

The width and height of the corresponding cross sections are given in cm

### Climate data

To examine moisture distributions, which are likely to occur in the future, corresponding weather data are required. We chose 2016 as an appropriate time period to obtain the RH and temperature data. This year was characterized by temperatures above average as well as ten percent more precipitation [28] and was selected because temperature and precipitation are expected to increase in the future [29]. The RH and temperature data over a time span of 14 months (November 1, 2015 until December 31, 2016), and for 6-hour intervals (3 a.m., 9 a.m., 3 p.m. and 9 p.m.), obtained from [30], are applied step-by-step on all outer surfaces as Neumann boundary conditions. The 6-hour interval was chosen due to data availability. Dietsch et al. [8] pointed out that the most significant moisture changes occur during the first winter and after assembly. Thus, the investigated period begins with November 1, 2015.

**Fig. 3:**
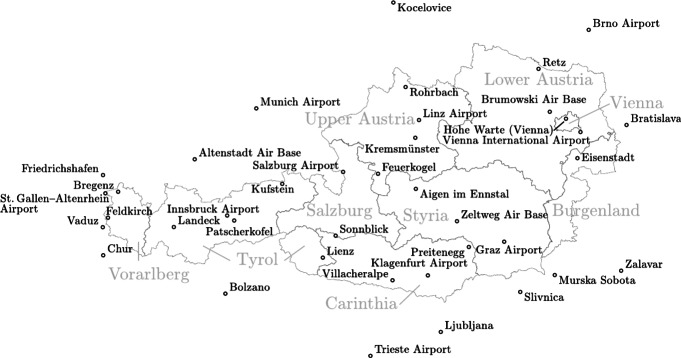
Overview of all weather stations, from which climate data were obtained

Climate data were obtained from 39 different weather stations, with approximate locations shown in Fig. 3. The number of weather stations is based on the availability of weather data for the 6-h intervals. The name of the weather station is related to their position, where 25 are located in Austria. The additional 14 are added to enable data interpolation throughout the Austrian territory. The weather stations are located at different sea levels above the Adriatic ranging between 11 m (Trieste Airport) and 3100 m (Sonnblick: mountain in Austria).

The moisture distributions to be determined for each cross section at all locations form the basis for calculating moisture fields covering Austria’s whole territory, enabling a location-specific moisture analysis. The software Matlab R2022a [31] is used to interpolate moisture simulation results from all locations, creating a value field over Austria. Comparing the climate data reveals significant RH and temperature differences between mountainous and non-mountainous weather stations. As we cannot estimate the climate between these points sufficiently, only a linear interpolation method is used for the generation of the moisture fields.

To compare the generated moisture field results with the standard ONR CEN/TS 19103 [7], additional values are introduced. $u_{\text{avg,cs}}$ defines the *u* averaged over the whole cross section, while $u_{\text{avg,BC}}$ describes *u*, which is averaged over the path from B to C (see Fig. 2). $\text{A}^{\text{max}}_{u_{\text{avg,cs}}}$ and $\text{A}^{\text{max}}_{u_{\text{avg,BC}}}$ are the maximum amplitudes of $u_{\text{avg,cs}}$ and $u_{\text{avg,BC}}$, respectively, which can be determined as:

2 $$\begin{aligned} \text{A}^{\text{max}}_{u_{\text{avg,cs}}}&= \text{max} \left(u_{\text{avg,cs}}\right) - \text{min} \left(u_{\text{avg,cs}}\right) \\ \text{A}^{\text{max}}_{u_{\text{avg,BC}}}&= \text{max} \left(u_{\text{avg,BC}}\right) - \text{min} \left(u_{\text{avg,BC}}\right). \end{aligned}$$

In addition, the influence of the altitude and the location on moisture content gradients is analyzed, where the definition of the gradient is based on Brandstätter et al.  [9]. They studied the relation between the crack depth development and the moisture gradient, which was introduced as the difference in *u* between the points B and C (see Fig. 2) divided by their distance $(\Delta u / \Delta x)_{\text{BC}}$. As the maximum gradients are related to the largest moisture-induced stresses, and, therefore, to the deepest cracks, the maximum gradient $(\Delta u / \Delta x)^{\text{max}}_{\text{BC}}$ is studied.

## Results and discussion

The following section shows the development of *u* over 14 months for two different locations along the path from B to C (see Fig. 2), exemplified for the ST $5\times 8$. In addition, the number of weeks, in which $u_{\text{avg,cs}}$ continuously exceeds 12% and 20%, respectively, and the distribution of moisture content gradients in Austria are illustrated. Furthermore, the results are compared with EC 5 [1] and EC 1 [32] as well as with the values of the standard ONR CEN/TS 19103 [7]. Besides, the points in time, when the maximum gradient $(\Delta u / \Delta x)^{\text{max}}_{\text{BC}}$ occur, are examined.

**Fig. 4:**
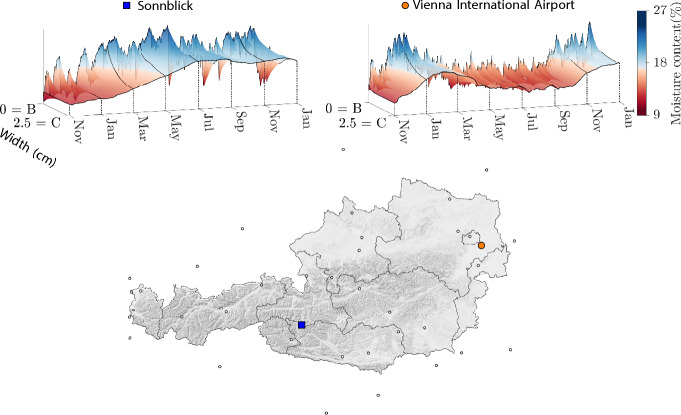
Relief map of Austria, including the weather station’s locations. In addition, moisture fluxes for the cross section ST $5\times 8$ (width $\times$ height, given in cm) along the path from B to C (see Fig. 2) over 14 months are shown, exemplified for the locations Sonnblick and Vienna International Airport

At first, a relief map of Austria is shown in Fig. 4 to provide a visual understanding of Austria’s geography and topography. To highlight the influence of the location and the altitude on the moisture content behavior, the moisture fluxes along the Path from B to C (see Fig. 2) for the cross section ST 5 $\times$ 8 over 14 months are exemplified for the locations Sonnblick and Vienna International Airport. Sonnblick and Vienna International Airport are chosen to optimally display this effect, as both locations differ enormously in topography, distance and altitude. Whereas the weather station at Vienna International Airport is positioned in the Vienna Basin in the northeast of Austria at 183 m sea level, the Sonnblick weather station is in the Alps in the southwest of Austria at 3100 m sea level.

### Location-specific moisture content developments

**Fig. 5:**
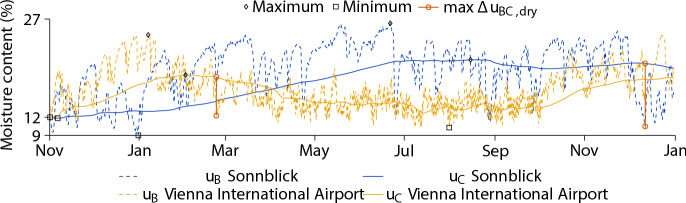
Moisture content development for the cross section ST $5 \times 8$ (width $\times$ height, given in cm) at the boundary B ($u_{\text{B}}$) and the center C ($u_{\text{C}}$; see Fig. 2), including their maxima and minima. In addition, the maximum difference between $u_{\text{C}}$ and $u_{\text{B}}$ in case of drying ($\Delta \,u_{\text{BC,dry}}$) is highlighted

The analysis gives the distribution of moisture content over the cross section and time, for each series of climate input. Figure 5 shows the resulting development in *u* at the boundary (B) and the center (C), exemplified for the ST $5 \times 8$. While $u_{\text{B}}$ and $u_{\text{C}}$ define the moisture content at the points B and C, respectively, $\Delta \,u_{\text{BC,dry}}$ is the difference between $u_{\text{C}}$ and $u_{\text{B}}$ in case of drying. The moisture distribution at Sonnblick is different from the results at Vienna International Airport in several points. To illustrate the influence of the ambient climate, the differences in $u_{\text{C}}$ for the ST $5 \times 8$ are examined. While the weather at Sonnblick is rather dry from November to January, resulting in a maximum $u_{\text{C}}$ of about 12.8%, the climate at Vienna International Airport is characterized by high RH, which is why $u_{\text{C}}$ increases to a maximum of 16.8% for the same time period. With the end of January, the RH at Sonnblick starts to increase and remain at a higher level, leading to an increase of $u_{\text{C}}$. In July, a peak is reached with a maximum of approximately 20.7%, which will not decrease significantly until September. In contrast, at Vienna International Airport, the RH and, therefore, $u_{\text{C}}$ decreases with February, and will not increase significantly until October, with a minimum of about 12.4% in July.

In addition, it can be seen that the total differences of the minimum $u_{\text{C}}$ for both locations is smaller than one percent, whereas the minimum difference in $u_{\text{B}}$ is about 1.2%. While the difference between the maximum $u_{\text{C}}$ of Sonnblick and Vienna International Airport is 2.4%, the maximum $u_{\text{B}}$ values vary by about 1.8%. The biggest difference is in the maximum of $\Delta \,u_{\text{BC,dry}}$, where the value of Sonnblick (9.7%) is one-and-a-half times the value at Vienna International Airport (6.0%). Also, the points in time of the maxima and minima of $u_{\text{B}}$, $u_{\text{C}}$ and $u_{\text{BC,dry}}$ vary depending on the location. While the maximum of $\Delta \,u_{\text{BC,dry}}$, and thus, the greatest chance for cracking at Sonnblick is given in December, at Vienna International Airport, it is in February.

### Distribution of the average moisture content in Austria

**Fig. 6:**
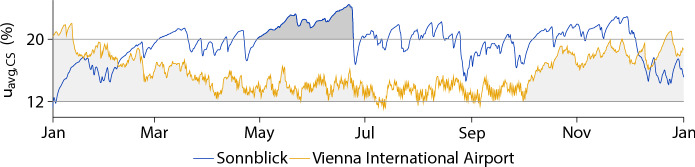
Development of the moisture content *u* averaged over the cross section $u_{\text{avg,cs}}$, exemplified for the cross sections ST $5 \times 8$ (width $\times$ height, given in cm) at the locations Sonnblick and Vienna International Airport. The filled dark-grey area highlights the period, in which $u_{\text{avg,cs}}$ continuously exceeds 20% for the longest time. The sum of the light grey areas shows just that for 12% moisture content

**Fig. 7:**
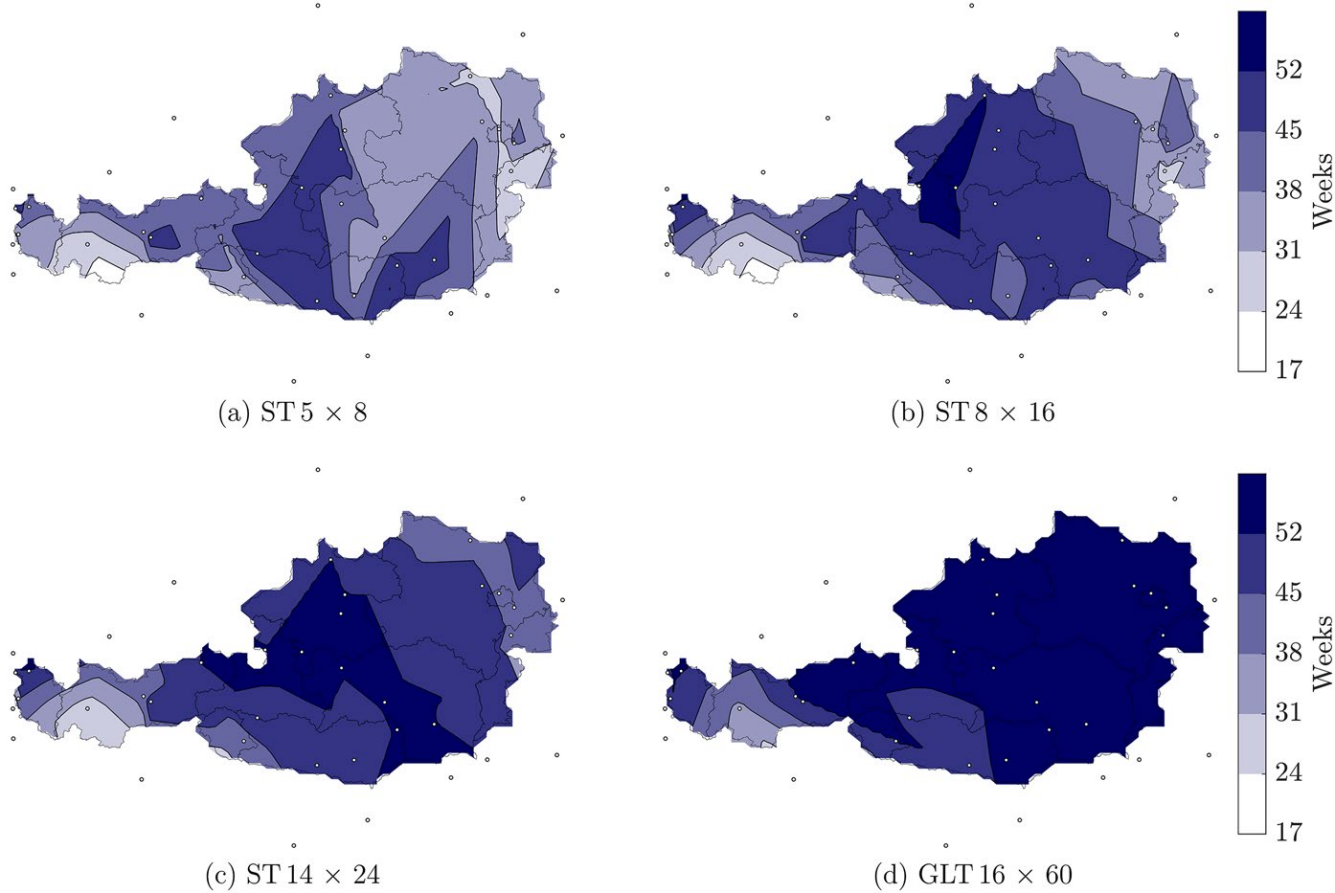
Number of weeks in which the average wood moisture content continuously exceeds 12% for the cross sections **a** ST $5\times 8$, **b** ST $8 \times 16$, **c** ST $14 \times 24$ and** d** GLT $16 \times 60$ (width $\times$ height, given in cm)

**Fig. 8:**
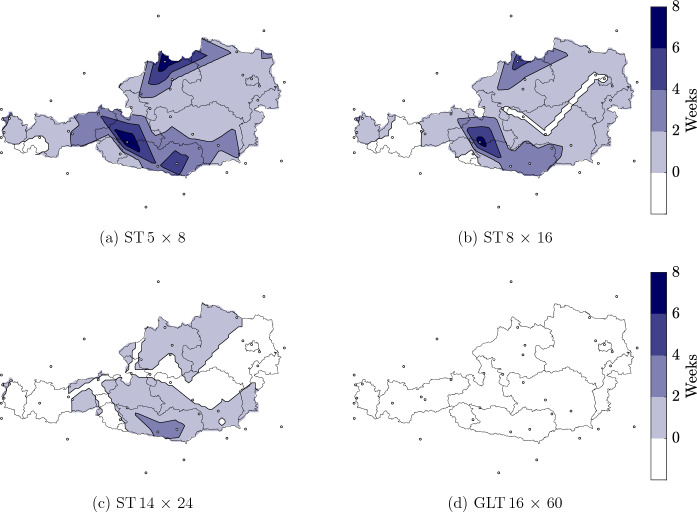
Number of weeks in which the average wood moisture content continuously exceeds 20% for the cross sections **a** ST $5 \times 8$, **b** ST $8 \times 16$, **c** ST $14 \times 24$ and **d** GLT $16 \times 60$ (width $\times$ height, given in cm)

The differences in location and altitude cause similar effects in $u_{\text{avg,cs}}$, which can be used as thresholds (12% and 20%) to assign service classes according to EC 5 [1]. Figure 6 shows the development of $u_{\text{avg,cs}}$, exemplified for the cross section ST $5\times 8$ at Sonnblick and at Vienna International Airport between January 1, 2016, and January 1, 2017. For both locations, $u_{\text{avg,cs}}$ is often between 12 and 20%, but in the case of the location Sonnblick, $u_{\text{avg,cs}}$ surpasses 20% for several months, whereas for Vienna International Airport, $u_{\text{avg,cs}}$ is less than 20% most of the year and even drops below 12% multiple times. It can be seen that due to the influence of the altitude and the location, different assignments to a service class should be made. Figure 7 shows the maximum number of weeks, in which $u_{\text{avg,cs}}$ continuously exceeds 12% (see light grey areas in Fig. 6), and Fig. 8 displays just that for 20% (see dark grey area in Fig. 6) for several cross sections. As $u_{\text{avg,cs}}$ can also exceed the thresholds over the turn of a year, the corresponding periods after the beginning of 2016 and at the of end 2016 (light grey areas) are added up.

Figure 7a–c shows that in case of the ST cross sections, regional differences are more pronounced compared to the GLT cross section GLT $16 \times 60$ (see Fig. 7d). Comparing all shown cross sections reveals that the number of weeks increases with the width of the cross section. While for the ST $5 \times 8$, the maximum number of weeks ranges between 17 and 52 weeks, for the GLT $16 \times 60$, the maximum number of weeks is between 31 and 52 weeks. This is related to the faster adaption of the moisture field to the environmental conditions of smaller cross sections, which is why $u_{\text{avg,cs}}$ falls below 12% more often for the ST cross sections, resulting in fewer weeks that are continuously above 12%. As for the cross section GLT $16 \times 60$ with the exception of areas in Carinthia, Salzburg, Styria, Tirol and Vorarlberg, $u_{\text{avg,cs}}$ exceeds 12% throughout the entire year. The maps illustrating the moisture behavior of GLT $20 \times 60$ and GLT $24 \times 60$ are not displayed due to minimal result variations compared to GLT $16 \times 60$. Besides, a comparatively large number of weeks continuously exceeding 12% $u_{\text{avg,cs}}$ can be seen in mountain regions (Feuerkogel, Patscherkofel, Sonnblick and Villacheralpe), which shows the influence of the altitude. The larger the cross section, the more weeks $u_{\text{avg,cs}}$ continuously exceeds 12%, particularly in the mountains.

Figure 8a–c show regions of Austria, in which $u_{\text{avg,cs}}$ continuously exceeds 20% for the ST cross sections. For at least one week, $u_{\text{avg,cs}}$ exceeds 20% in case of the cross section ST $5 \times 8$ (except in parts of Tirol and Vorarlberg), where the maxima can be located at Klagenfurt Airport, Rohrbach and Sonnblick (see Fig. 8a). In case of the cross sections ST $8 \times 16$ and ST $14 \times 24$, the zones exceeding a $u_{\text{avg,cs}}$ of 20% shrink, where the maxima appear at the same locations as for the ST $5 \times 8$ (see Fig. 8b and c). While for the ST $8 \times 16$ in parts of Lower Austria, Styria, Tirol, Upper Austria, Vienna and Vorarlberg $u_{\text{avg,cs}}$ stays below 20%, in case of the ST $14 \times 24$ in areas of Burgenland and Salzburg at no time $u_{\text{avg,cs}}$ is above 20% additionally. It can be seen, that with increasing cross section size the maximum of $u_{\text{avg,cs}}$ decreases, and, thus, values above 20%. In addition, with increasing cross section size, less maxima of $u_{\text{avg,cs}}$ can be seen at non-mountainous regions. Thus, for smaller cross sections, a more significant effect of the location can be assumed. For the GLT cross sections, only one map is displayed (see Fig. 8d), since $u_{\text{avg,cs}}$ is always below this limit due to the slower adaption of the moisture field.

Depending on the location and the cross section size, $u_{\text{avg,cs}}$ can exceed 20%. Thus, the results indicate that cross sections with a size similar to or smaller than ST $14 \times 24$, should be assigned to service class 3. Besides, Fig. 8a and b display $u_{\text{avg,cs}}$ larger than 20% *u* in Rohrbach for at least four weeks, which is the longest time compared to all other non-mountainous locations. According to EC 1 [32], the location around Rohrbach is characterized by a comparatively large snow load (Rohrbach: $6.5\,\text{kN/m}^2$, minimum: $0.6\,\text{kN/m}^2$), indicating a correlation between the RH level and the amount of snow.

### Distribution of moisture content gradient maxima

**Fig. 9:**
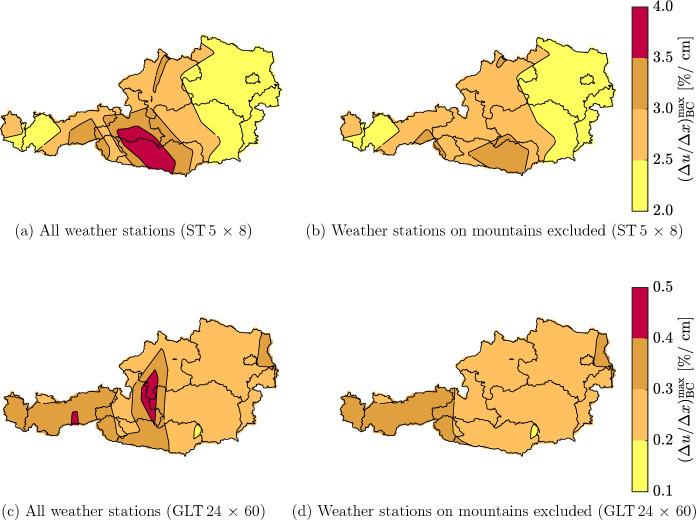
Distribution of the maximum moisture gradient $(\Delta u / \Delta x)^{\text{max}}_{\text{BC}}$ in Austria for the (**a** and **b**) ST $5 \times 8$ and (**c** and **d**) GLT $24 \times 60$ (width $\times$ height, given in cm). $(\Delta u / \Delta x)^{\text{max}}_{\text{BC}}$ is defined as the maximum difference in *u* between the points B and C (see Fig. 2) related to their distance

Figure 9 shows the distribution of the maximum gradient $(\Delta u / \Delta x)^{\text{max}}_{\text{BC}}$ in Austria for the ST $5\times 8$ (a and b) and GLT $24 \times 60$ (c and d). To demonstrate the influence of the altitude, Fig. 9b and d shows the distribution excluding weather stations on mountains (Feuerkogel, Patscherkofel, Sonnblick, and Villacheralpe), whereas Fig. 9a and c considers all weather stations. For the ST $5 \times 8$, it can be seen that due to larger moisture gradients, parts of Carinthia, Salzburg, Styria, Tyrol and Upper Austria are more prone to cracks than the rest of Austria. However, when the weather stations on the mountains are excluded, the maximum gradient decreases in some of the regions mentioned before. For the GLT $24 \times 60$, the same can be observed. When considering all weather stations, a maximum gradient up to 0.5%/cm can be reached in Carinthia, Salzburg, Styria, Tirol and Upper Austria, while without the maximum is 0.4%/cm. These differences demonstrate the significant influence of the altitude on the moisture gradient. Thus, weather data from locations close to mountains are required to improve the accuracy of the maps and reduce the error caused by mountain influence. However, it can be seen that the location also affects the maximum gradient. For the ST $5 \times 8$, $(\Delta u / \Delta x)^{\text{max}}_{\text{BC}}$ reaches up to 2.5%/cm in Burgenland, Lower Austria, Styria, Upper Austria and Vienna, while a maximum of 3.5%/cm can occur in Carinthia, Styria and Tyrol. For the GLT $24 \times 60$, the differences are less pronounced. Only in parts of Lower Austria, Salzburg, Tyrol and Vorarlberg, the maximum gradient is up to 0.3%/cm larger than in the other states. Thus, influence from the location on $(\Delta u / \Delta x)^{\text{max}}_{\text{BC}}$ is relevant, but decreases with cross section size. Therefore, the maps would also benefit from additional data of non-mountainous locations to increase the accuracy and quality, particularly for smaller cross sections.

### CEN/TS 19103

**Fig. 10:**
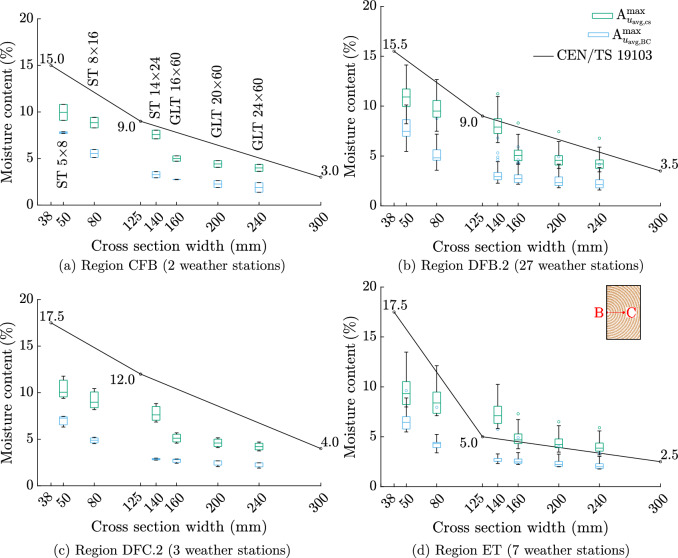
Comparison of the maximum amplitude of the moisture content averaged over the cross section $\text{A}^{\text{max}}_{u_{\text{avg,cs}}}$ of the standard ONR CEN/TS 19103 [7] and the simulations results as boxplots for the regions **a** CFB, **b** DFB.2, **c** DFC.2 and **d** ET [33]. In addition, the maximum amplitudes of the moisture content averaged over the path from B to C $\text{A}^{\text{max}}_{u_{\text{avg,BC}}}$ are shown

In the following section, the maximum amplitudes of *u* ($\text{A}^{\text{max}}_{u_{\text{avg,cs}}}$) obtained from the simulation results are compared to the values of the standard CEN/TS 19103, which defines common rules for the design of timber–concrete composite structures [7]. In annex A, CEN/TS 19103 specifies maximum yearly amplitudes for *u* averaged over the cross section (equal to $\text{A}^{\text{max}}_{u_{\text{avg,cs}}}$) depending on one of twelve Köppen–Geiger climatic regions [33] and on the cross section’s width (three different given). These amplitudes are reference points to consider deformations and stresses caused by variations in temperature and moisture content of timber–concrete composite structures and allow interpolation between the three mentioned widths (see Fig. 10). The weather data in this work can be attributed to four of the twelve climatic regions (see Table 2).

**Table 2:** Values of the maximum yearly moisture content amplitudes averaged over the cross section $\text{A}^{\text{max}}_{u_{\text{avg,cs}}}$ (%)

Climatic region	Cross section width (mm)			Number of weather stations per region
	38	125	$\ge 300$	
CFB	15.0	9.0	3.0	2
DFB.2	15.5	9.0	3.5	27
DFC.2	17.5	11.5	4.0	3
ET	17.5	5.0	2.5	7

The values are obtained from ONR CEN/TS 19103 [7] and depend on the Köppen–Geiger climatic region [33] and on the cross section’s width. In addition, the number of weather stations, which are attributed to which region, are shown

As for the data obtained from the weather stations, the results of the simulations are assigned accordingly and displayed for each climatic region and cross section in Fig. 10. In addition to the maximum amplitude ($\text{A}^{\text{max}}_{u_{\text{avg,cs}}}$), the values of the standard and $\text{A}^{\text{max}}_{u_{\text{avg,BC}}}$ [see Eq. (2)] are presented. It can be seen that in case of the regions CFB and DFC.2, the maximum amplitudes are always smaller than the ones of ONR CEN/TS 19103 [7], but since only data from maximum three weather stations are available, the results are less representative. In case of the regions DFB.2 and ET, the maximum amplitude is differently distributed compared to the other two regions. While for the cross sections ST $5 \times 8$ and ST $8 \times 16$, about 94% of the values are smaller than the interpolated ones of ONR CEN/TS 19103 [7], for larger cross sections, the maximum amplitude can be greater than the values of the standard. Whereas in case of the region DFB.2, 90% of the values are smaller than the interpolated ones of ONR CEN/TS 19103 [7], in case of the region ET, only 5% of the maximum amplitudes are smaller than the interpolated standard values. In addition, it must be emphasized that for cross sections with widths greater than 14 cm, the height to width ratio was larger compared to the smaller cross sections. If those would have a similar ratio as the smaller cross sections, i.e., ratios of about 1.7, it is assumed that at more locations the maximum amplitude would be larger than the values of ONR CEN/TS 19103 [7]. For the second investigated definition of an amplitude $\text{A}^{\text{max}}_{u_{\text{avg,BC}}}$, the simulation results are always lower than the standard values due to the absence in height influence on the amplitude. For the regions CFB, DFB.2 and DFC.2, all $\text{A}^{\text{max}}_{u_{\text{avg,BC}}}$ values are much smaller than the ones of the standard. Only in case of the region ET, the values of the cross sections with a width larger than 12.5 cm are close the ones of ONR CEN/TS 19103 [7].

In addition, comparing the distribution of the maximum gradients with the standard values of the maximum yearly moisture content amplitude $\text{A}^{\text{max}}_{u_{\text{avg,cs}}}$ reveals that at those locations, where the maximum gradients tend to occur, the maxima of $\text{A}^{\text{max}}_{u_{\text{avg,cs}}}$ are also found.

### Moisture content and moisture content gradient distribution

Additionally, the location-specific developments of *u* are analyzed. As shown in Fig. 5, for the ST $5 \times 8$, the maximum occurring difference in *u* between B and C in case of drying at Sonnblick is one-and-a-half times the value as at Vienna International Airport, caused by a humid phase beginning in spring and followed by a dry period starting in November. Since with greater differences in *u* between B and C in case of drying and moisture gradients, respectively, the maximum crack depth $d_{\text{c}}^{\text{max}}$ increases [9], the potential reduction of the load-bearing capacity of wooden structures at Sonnblick is higher than at Vienna International Airport.

**Fig. 11:**
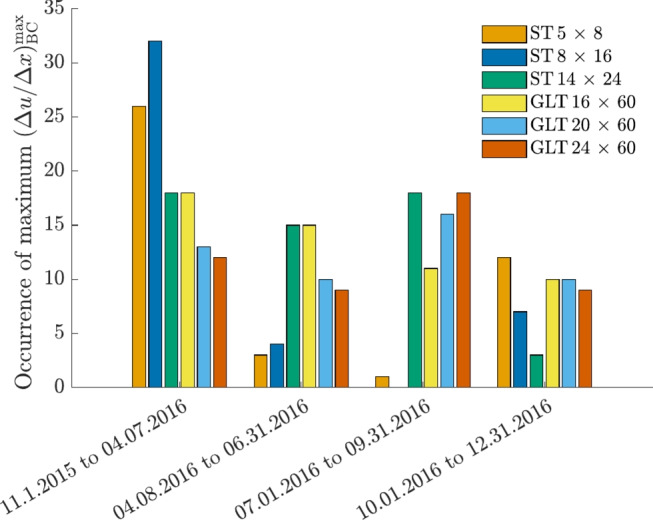
Distribution of how often the maximum moisture content gradient $(\Delta u / \Delta x)^{\text{max}}_{\text{BC}}$ occurs in the given periods. $(\Delta u / \Delta x)^{\text{max}}_{\text{BC}}$ is defined as the maximum difference in *u* between the points B and C (see Fig. 2) related to their distance

Due to the location-dependent climate, the maximum gradient $(\Delta u / \Delta x)^{\text{max}}_{\text{BC}}$ occurred at different points in time, as illustrated in Fig. 11. For non-mountainous locations, as the cross section size increases, more maxima can be observed between April and the end of September, whereas for smaller cross sections, the maximum gradient tends to occur between November and April. This was also observed by Autengruber et al. [3], as for greater cross sections (GLT $20 \times 40$) the maximum gradient was reached in August, and for the investigated ST $6 \times 8$, it occurred in March for the location Linz, Austria. Dietsch et al. [8] pointed out that the greatest differences in *u* often happen during the first winter. This accords with our findings. For locations in the mountains, most of the maximum gradients occurred in December. Maximum gradients occurring between October 1, and December 31, 2016, are related to locations in the mountains or the western part of Austria for all cross sections.

**Fig. 12:**
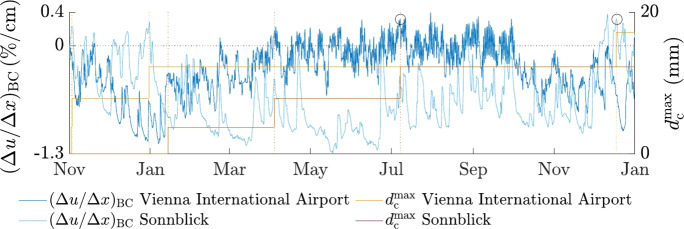
Development of the moisture gradient $(\Delta u / \Delta x)_{\text{BC}}$ and the maximum total crack depth $d_{\text{c}}^{\text{max}}$ for the cross section GLT $20\times 60$ (width $\times$ height, given in cm), exemplified for the locations Sonnblick and Vienna International Airport (VIE). $(\Delta u / \Delta x)_{\text{BC}}$ is defined as the difference in *u* between the points B and C related to their distance. The vertical dotted red and orange line help to perceive which events cause an increase in $d_{\text{c}}^{\text{max}}$ and the circles highlight the points in time of the moisture profiles illustrated in Fig. 13

The previously shown data and Fig. 9 highlight the influence of the location-dependent climate on the moisture gradient $(\Delta u / \Delta x)_{\text{BC}}$. Therefore, at specific locations for smaller cross sections and at high altitudes, larger moisture-induced stresses and, thus, more significant crack depths are expected. How deep cracks may propagate in wooden cross sections exposed to outdoor climate conditions, is analyzed in the following. To determine $d_{\text{c}}^{\text{max}}$ (sum of the deepest cracks at both the left and the right edge), additional XFEM simulations were performed (see Fig. 1). Figure 12b shows the resulting development of the moisture gradient and $d_{\text{c}}^{\text{max}}$ over the whole simulation period, exemplified for the GLT $20 \times 60$ at the locations Sonnblick and Vienna International Airport. For these locations, it can be observed that at peaks of the moisture gradient, $d_{\text{c}}^{\text{max}}$ tends to increase. However, in December 2016, for the location Sonnblick, e.g., the maximum of the moisture gradient occurred without any change in $d_{\text{c}}^{\text{max}}$. But, a closer look at the simulation results revealed that, at this point in time, high moisture gradients resulted in crack propagation, but not for the cracks with the largest depth, causing no increase in $d_{\text{c}}^{\text{max}}$.

**Fig. 13:**
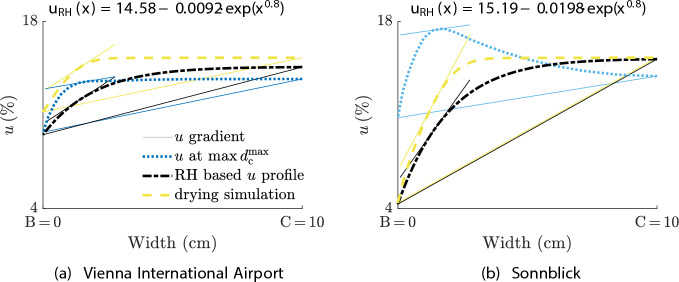
Moisture content (*u*) profiles for the cross section GLT $20 \times 60$ (width $\times$ height, given in cm), exemplified for the locations **a** Vienna International Airport and **b** Sonnblick. The dash-dotted line shows the lower *u* envelope curve from Autengruber et al. [17] only based on relative humidity (RH) data. The dashed line displays the distribution of *u* from the simulation results of Brandstätter et al. [9]. The dotted line shows the *u* profile at the point in time when the maximum total crack depth $d_{\text{c}}^{\text{max}}$ occurs (see black circles in Fig. 12). The continuous lines illustrate two different moisture gradient configurations for all *u* profiles

To avoid moisture and XFEM simulations lasting several days, efforts were made to estimate $d_{\text{c}}^{\text{max}}$ only based on RH data of the investigated time span in case of drying. Therefore, the results from Autengruber et al. [17] and Brandstätter et al. [9] were combined, and verified with the results of this work. In Fig. 13, the approach is visualized for the cross section GLT $20\times 60$ and exemplified for the locations Vienna International Airport and Sonnblick. Cracks are the result of immense stresses, which are induced by constrained volume changes caused by non-uniform moisture distributions. Brandstätter et al. [9] showed that instead of moisture fields, moisture gradients evaluated along the path from B to C (see Fig. 2) suffice to describe the development of $d_{\text{c}}^{\text{max}}$ quantitatively. With the equation from Autengruber et al. [17] and sufficient RH data, one can estimate the lower *u* envelope curve from B to C in case of drying (see dash-dotted line in Fig. 13). The lower *u* envelope curve was assumed to be the most severe drying case and, therefore, result in a $d_{\text{c}}^{\text{max}}$ being equal to or minimally larger than the maximum $d_{\text{c}}^{\text{max}}$ from this work’s XFEM simulation (verification value). The equation from Autengruber et al. [17] reads as follows:

3 $$\begin{aligned} u(x) = u_{\text{C}} - F_{\text{dist}} \exp \left(x^{0.8}\right), \end{aligned}$$

with *x* as the distance from C in cm, and $F_{\text{dist}}$ as the exponential distribution factor. $u_{\text{C}}$ can be determined based on the averaged RH data and the adsorption isotherm, and $F_{\text{dist}}$ by calculating $u(x=\text{B})$ (see Autengruber et al. [17] for detailed determination information). Brandstätter et al. [9] simulated the development of *u* for various RH reductions and initial *u* levels over time (drying simulations), including the evolution of $d_{\text{c}}^{\text{max}}$ resulting from these drying loads. From these drying simulations, we determined the *u* profile characterized by the smallest moisture gradient error to the lower *u* envelope curve (see dashed and dash-dotted lines in Fig. 13). The smallest moisture gradient error is the minimum difference in the moisture gradient $(\Delta u / \Delta x)_{\text{BC}}$ and another moisture gradient evaluated approximately 15 mm distant to B. The consideration of an additional moisture gradient configuration was necessary to unambiguously determine the corresponding *u* profile, and from the introduced moisture gradient configurations from Brandstätter et al. [9], the one chosen provided the most accurate results. Based on the *u* profile of the drying simulation, the corresponding $d_{\text{c}}^{\text{max}}$ was derived, which was compared to the maximum $d_{\text{c}}^{\text{max}}$ determined from this work’s XFEM simulation to verify the approach. However, the derived maximum $d_{\text{c}}^{\text{max}}$ were up to four times larger than the ones of this work. The reason for this can be seen when the moisture gradients of the lower *u* envelope curve (and of the *u* profile from the drying simulations, respectively) are compared to the ones of the *u* profile resulting from the climate data. For Vienna International Airport, the moisture gradient $(\Delta u / \Delta x)_{\text{BC}}$ hardly varies, but a significant difference can be seen for the other moisture gradient configuration. For Sonnblick, both moisture gradient configurations differ notably. The stress states caused by these moisture gradient configurations vary considerably, therefore, the resulting $d_{\text{c}}^{\text{max}}$ are not sufficiently equal. If moisture simulations based on the climate data are performed and the moisture gradients determined at the point in time when the maximum gradient occurs are used to find the smallest moisture gradient error within the drying simulations, the maximum $d_{\text{c}}^{\text{max}}$ is still up to twice as large as the verification value. The maximum moisture gradients resulting from the climate data and the derived values of Brandstätter et al. [9] differ significantly due to different boundary conditions and investigation periods. While the climate’s RH history with numerous weather cycles over 14 months causes multiple drying and moistening phases, the drying simulation is only characterized by a single event, where the surrounding RH is reduced. Therefore, the difference in the moisture gradients suffices to cause an inaccurate estimation of $d_{\text{c}}^{\text{max}}$. Only at the beginning of the simulation does an estimation of $d_{\text{c}}^{\text{max}}$ seem possible due to minimal differences, but it is insufficient as larger moisture gradients are very likely to occur later.

The *u* profiles from the locations Sonnblick and Vienna International Airport (see dotted lines in Fig. 13) represent the occurring moisture state, when $(\Delta u / \Delta x)_{\text{BC}}$ reaches its maximum: starting from C to B, *u* decreases continuously (Vienna International Airport), and *u* increases at one point and then decreases again (Sonnblick). For the GLT $20\times 60$, 29 of the 39 investigated locations have an *u* profile like that at Sonnblick. With decreasing cross section size, the *u* profiles differ from the one resembling Sonnblick’s profile. While for the ST $14 \times 24$, 20 locations can be attributed to the profile of Sonnblick, for the ST $5 \times 8$, only six are alike the development of Sonnblick. The profiles of *u* were analyzed for ST $5 \times 8$, ST $14 \times 24$ and GLT $20 \times 60$ due to the resemblance of the cross sections examined in Brandstätter et al. [9].

## Conclusion and outlook

The present study examined the moisture content (*u*) developments of three ST and three GLT cross sections at 39 locations in and around Austria over 14 months based on numerical simulations. To simulate moisture transport, a multi-Fickian model was used [10–15]. Subsequently, XFEM simulations were performed to determine moisture-induced stresses, which may lead to cracks. A multisurface failure criterion from [21–24] defined the strength limit of wood, and with the results of Hofstetter et al. [20], moisture-dependent material properties were considered. Based on the simulation results, the development of *u* averaged over the cross section $u_{\text{avg,cs}}$ and the maximum moisture gradient $(\Delta u / \Delta x)^{\text{max}}_{\text{BC}}$ distribution in Austria were analyzed. The results were compared with the standards EC 1 [32], EC 5 [1] and ONR CEN/TS 19103 [7], which defines common rules for the design of timber–concrete composite structures. In addition, the point in time when $(\Delta u / \Delta x)^{\text{max}}_{\text{BC}}$ occurred and, thus, potential crack developments were investigated.

The main conclusions can be summarized as follows:

- For smaller cross sections, a significant influence of both location and altitude on the evolution of *u* can be seen. However, with increasing cross sections size the influence of the location on the development of *u* decreases, and primarily the altitude affects *u*.
- The location and especially the altitude also affect the occurrence and magnitude of the maximum moisture content gradient $(\Delta u / \Delta x)^{\text{max}}_{\text{BC}}$. While $(\Delta u / \Delta x)^{\text{max}}_{\text{BC}}$ of smaller cross sections (ST $5\times 8$ and ST $8 \times 16$) is reached between the beginning of the investigation period (November 1, 2015) and the middle of April, with increasing cross section size, $(\Delta u / \Delta x)^{\text{max}}_{\text{BC}}$ could be observed between the middle of April and the end of September.
- In addition, larger moisture content gradients $(\Delta u / \Delta x)^{\text{max}}_{\text{BC}}$ are found at higher altitudes, indicating that deeper cracks likely occur in these regions.
- The standard CEN/TS 19103 introduces values for the maximum yearly moisture content amplitudes averaged over the cross section $\text{A}^{\text{max}}_{u_{\text{avg,cs}}}$. Comparing these with our results reveals appropriate values in most cases. Only for cross sections with a width of 14 cm and larger assigned to the climate region ET, $\text{A}^{\text{max}}_{u_{\text{avg,cs}}}$ is assumed to be underestimated.
- The maximum resulting crack depth estimated only based on RH data could not be sufficiently predicted. The RH data were used to estimate the lower *u* envelope curve, which was assumed to cause the most significant stresses and, therefore, the deepest cracks. From the simulation results of Brandstätter et al. [9], the *u* profile with the smallest moisture gradient error to the lower *u* envelope curve was determined. The maximum crack depth resulting by this *u* profile was compared to the deepest cracks determined with the RH data within the framework of an XFEM simulation. However, the estimated maximum crack depth was up to four times larger than the one from the XFEM simulation.

Additional simulations with climate data from different years are recommended to confirm the presented findings and distinguish the individual case from the regular case. Besides, weather data from more locations would improve the accuracy of the maps. Particularly climate data from locations close to mountains are essential to reduce the error caused by mountain influence.

By using the simulation tool, coupled with extensive climate data, we generated moisture fields for several cross sections at 39 locations over 14 months, a significant advancement in detailed data quantity. Austria was used as an example, as many different climatic conditions occur. With the appropriate amount of data, such simulations can be performed for other countries or climatic regions.

In addition, it seems reasonable to estimate possible future climate conditions and perform moisture simulations to anticipate unfavorable situations.

Finally, it is also worth noting that beyond geographical location, local conditions such as the micro-climate and “micro-boundary conditions” can significantly influence the distribution of *u*.

## Supplementary Information

Supplementary Material 1.

## Data Availability

The data used and/or analyzed during the current study are available from the corresponding author on reasonable request.

## Abbreviations

$(\Delta u / \Delta x)^{\text{max}}_{\text{BC}}$: Maximum of $(\Delta u / \Delta x)_{\text{BC}}$ [%/cm]
$(\Delta u / \Delta x)_{\text{BC}}$: Moisture gradient, which can be defined as the difference in the moisture content between the points B and C (see Fig. 2) divided by their distance [%/cm]
$d_{\text{c}}^{\text{max}}$: Sum of the deepest cracks at both the left and the right edge, defined as the maximum total crack depth [mm]
$\text{A}^{\text{max}}_{u_{\text{avg,BC}}}$: Maximum amplitude of the moisture content averaged over the path from B to C (see Fig. 2) [%]
$\text{A}^{\text{max}}_{u_{\text{avg,cs}}}$: Maximum amplitude of the moisture content averaged over the cross section (cs) [%]
*u*: Moisture content [%]
$u_{\text{avg,BC}}$: Moisture content averaged over the path from B to C (see Fig. 2) [%]
$u_{\text{avg,cs}}$: Moisture content averaged over the cross section [%]
GLT: Glued laminated timber
MUF: Melamine urea-formaldehyde
ST: Solid timber
ST $5\times 8$: Solid timber cross section, with a width of 5 cm and a height of 8 cm
UF: Urea-formaldehyde
XFEM: Extended finite element method
RH: Relative humidity [%]
