# Supplementary material for: The influence of geographical location on moisture distribution in wood cross sections: a numerical simulation study using Austria as an example
Source: J Wood Sci. 2024 Jul 30;70(1):35. doi: 10.1186/s10086-024-02147-z (PMC11385801; doi:10.1186/s10086-024-02147-z)
Supplement: Supplementary file 1 — Supplementary Material 1. [file 10086_2024_2147_MOESM1_ESM.pdf]

Supplematry material of The influence of geographical location on moisture distribution in wood cross sections: a numerical simulation study using Austria as an example

Florian Brandstätter[1*], Maximilian Autengruber[1], Markus Lukacevic[1] and Josef Füssl[1]

[1*]TU Wien, Institute for Mechanics of Materials and Structures, Karlsplatz 13, Vienna, 1040, Austria.

*Corresponding author(s). E-mail(s): florian.brandstaetter@tuwien.ac.at;

Expansion coefficients

Table S1 Expansion coefficients α for temperature [1] and β for moisture [2]

	α [1/K]	β [%/%]
L	3.15 E-6	0.015
R	23.8 E-6	0.19
T	32.3 E-6	0.36

Elasticity tensor of spruce

Table S2 Tensor components C_{iiii} [MPa] for spruce elasticity at 293.15 K with a dry density of 420 kg/m^3 [3].

u [%]	C_{LLLL}	C_{RRRR}	C_{TTTT}	C_{LLRR}	C_{RRTT}	C_{TTLL}	C_{LRLR}	C_{LTLT}	C_{RTRT}
3	13981.39	1127.15	755.30	343.76	272.01	520.26	396.68	389.40	53.70
4	13824.31	1094.04	733.20	337.33	266.94	504.70	385.64	378.57	52.23
5	13669.80	1061.40	711.42	330.88	261.87	489.33	374.67	367.80	50.79
6	13517.80	1029.22	689.96	324.41	256.78	474.16	363.78	357.11	49.37
7	13368.23	997.51	668.82	317.92	251.67	459.21	352.97	346.49	47.97
8	13221.06	966.26	647.98	311.40	246.55	444.47	342.25	335.97	46.60
9	13076.22	935.48	627.46	304.86	241.40	429.95	331.63	325.55	45.24
10	12933.66	905.17	607.25	298.29	236.23	415.66	321.12	315.23	43.91
11	12793.34	875.33	587.34	291.68	231.03	401.60	310.72	305.02	42.59
12	12655.21	845.95	567.74	285.04	225.80	387.77	300.45	294.94	41.29
13	12519.23	817.05	548.45	278.37	220.55	374.19	290.31	284.99	40.00
14	12385.35	788.61	529.47	271.66	215.27	360.84	280.32	275.18	38.73
15	12253.54	760.65	510.79	264.92	209.95	347.74	270.47	265.51	37.47
16	12123.75	733.16	492.42	258.14	204.61	334.88	260.78	256.00	36.23
17	11995.96	706.15	474.35	251.33	199.24	322.26	251.25	246.64	35.01
18	11870.13	679.60	456.59	244.48	193.84	309.90	241.89	237.46	33.79
19	11746.22	653.53	439.14	237.61	188.41	297.77	232.71	228.44	32.60
20	11624.20	627.93	421.99	230.71	182.96	285.89	223.70	219.60	31.41
21	11504.05	602.80	405.15	223.78	177.49	274.26	214.88	210.94	30.24
22	11385.74	578.14	388.62	216.82	171.99	262.86	206.25	202.47	29.08
23	11269.23	553.95	372.39	209.85	166.48	251.71	197.81	194.18	27.94
24	11154.50	530.22	356.46	202.86	160.95	240.79	189.56	186.09	26.81
25	11041.52	506.95	340.83	195.86	155.41	230.11	181.51	178.18	25.70
26	10930.28	484.14	325.51	188.86	149.86	219.66	173.66	170.47	24.60
27	10820.74	461.80	310.49	181.85	144.30	209.44	166.00	162.96	23.52
28	10712.87	439.90	295.77	174.84	138.75	199.44	158.55	155.64	22.45
29	10606.66	418.46	281.35	167.84	133.20	189.67	151.29	148.52	21.40
30	10502.09	397.46	267.22	160.84	127.65	180.11	144.23	141.59	20.36

References

[1] Weatherwax RC, Stamm AJ (1956) The coefficients of thermal expansion of wood and wood products. Technical Report 1487, US Forest Products Laboratory

[2] Gloimüller S, De Borst K, Bader T, Eberhardsteiner J (2012) Determination of the linear elastic stiffness and hygroexpansion of softwood by a multilayered unit cell using poromechanics. Interaction and multiscale mechanics 5(3): 229–265

[3] Hofstetter K, Hellmich C, Eberhardsteiner J (2005) Development and experimental validation of a continuum micromechanics model for the elasticity of wood. European Journal of Mechanics - A/Solids 24(6): 1030–1053
